# Genome‐wide association mapping of Hagberg falling number, protein content, test weight, and grain yield in U.K. wheat

**DOI:** 10.1002/csc2.20692

**Published:** 2022-03-04

**Authors:** Jon White, Rajiv Sharma, David Balding, James Cockram, Ian J. Mackay

**Affiliations:** ^1^ Genetics and Breeding Dep. NIAB 93 Lawrence Weaver Road Cambridge, CB3 0LE UK; ^2^ Institute of Genetics Univ. College London London, WC1E 6BT UK; ^3^ Scotland's Rural College (SRUC) Kings Buildings, West Mains Road Edinburgh, EH9 3JG UK; ^4^ Current address: Melbourne Integrative Genomics Univ. of Melbourne Melbourne Australia

## Abstract

Association mapping using crop cultivars allows identification of genetic loci of direct relevance to breeding. Here, 150 U.K. wheat (*Triticum aestivum* L.) cultivars genotyped with 23,288 single nucleotide polymorphisms (SNPs) were used for genome‐wide association studies (GWAS) using historical phenotypic data for grain protein content, Hagberg falling number (HFN), test weight, and grain yield. Power calculations indicated experimental design would enable detection of quantitative trait loci (QTL) explaining ≥20% of the variation (PVE) at a relatively high power of >80%, falling to 40% for detection of a SNP with an *R^2^* ≥ .5 with the same QTL. Genome‐wide association studies identified marker‐trait associations for all four traits. For HFN (*h* ^2 ^= .89), six QTL were identified, including a major locus on chromosome 7B explaining 49% PVE and reducing HFN by 44 s. For protein content (*h* ^2 ^= 0.86), 10 QTL were found on chromosomes 1A, 2A, 2B, 3A, 3B, and 6B, together explaining 48.9% PVE. For test weight, five QTL were identified (one on 1B and four on 3B; 26.3% PVE). Finally, 14 loci were identified for grain yield (*h* ^2 ^= 0.95) on eight chromosomes (1A, 2A, 2B, 2D, 3A, 5B, 6A, 6B; 68.1% PVE), of which five were located within 16 Mbp of genetic regions previously identified as under breeder selection in European wheat. Our study demonstrates the utility of exploiting historical crop datasets, identifying genomic targets for independent validation, and ultimately for wheat genetic improvement.

## Core Ideas

·An association mapping panel of 150 genotyped U.K. winter wheats was assembled.
·Data for four grain quality traits was sourced from cultivar registration records.
·Genome‐wide association studies (GWAS) identified at least five QTL for each trait.
·A major GWAS hit for Hagberg falling number was identified on chromosome 7A.
·A third of yield GWAS hits co‐located with genetic loci under breeder selection.

**Abbreviations**
GWAS: genome‐wide association study
HFN: Hagberg falling number
HGCA: Home Grown Cereals Authority
LD: linkage disequilibrium
MAF: minor allele frequency
PCA: principal component analysis
PHS: preharvest sprouting
QTL: quantitative trait locus
SNP: single nucleotide polymorphism.

## 1 INTRODUCTION

Bread wheat (*Triticum aestivum* L.) is the world's most widely cultivated cereal crop, with global demand projected to increase by 60% by 2050. Breeding over the last 100 years has been very successful in increasing wheat yields (e.g., Mackay et al., 2021), and the most sustainable way to meet increased future demand is via faster genetic improvement. Accordingly, the development of high yielding wheat cultivars with good bread‐making quality is a major focus for wheat breeders (Reif et al., 2011). Over recent decades, breeding has resulted in steady genetic gains in European wheat (Mackay et al., 2011). With the aim of further increasing the rates of wheat genetic improvement, the incorporation of marker‐assisted strategies into breeding pipelines is now common. In marker‐assisted selection (MAS) approaches, genetic loci must first be identified, and linked or diagnostic markers developed (Adamski et al., 2020). Historically, biparental mapping populations have predominantly been used for genetic mapping in wheat. A disadvantage of such approaches is that such populations capture limited genetic variation, and that the effects of quantitative trait loci (QTL) can be overestimated (Cockram & Mackay, 2018). The use of these QTL in marker‐assisted selection often does not give expected results, as the biparental populations investigated may not represent breeding germplasm or may not segregate for the targeted QTL (Cockram & Mackay, 2018; Kulwal et al., 2012). More recently, association mapping, first developed for human genetics (Bodmer, 1986; Welcome Trust Case Control Consortium, 2007), has been used for genetic analysis in cereal crops (e.g., Bentley et al., 2014; Cockram et al., 2008, 2010; Corsi et al., 2020; Mellers et al., 2020; Waugh et al., 2010). These capture high levels of genetic diversity and genetic recombination (Cavanagh et al., 2008). Additionally, historical phenotypic data is often available (Mackay et al., 2011). A useful source of historical traits of relevance to growers and users are those collected during the process of cultivar registration (Jamali et al., 2019). In the United Kingdom, four such traits of agronomic or end‐use importance are routinely measured during the process of wheat cultivar registration for the Agriculture and Horticulture Development Board Recommended List: grain yield, protein content, Hagberg falling number (HFN), and test weight (also known as ‘specific weight’).

Grain yield is the principle agronomic trait for wheat breeders and growers. Yield is often found to be of low to moderate heritability, although it shows clear evidence for genetic improvement—for example in U.K. wheat yields over a period of 30 years (Mackay et al., 2011). However, historical datasets however often cover a far broader range of cultivar yields, with each cultivar also tested over several years at many locations, with a consequential and substantial increase in heritability. However, the highly polygenic nature of yield means identification of QTL remains challenging. At a fundamental level, grain yield can be described using three major factors: grain size (e.g., grain length, width, weight, shape), the numbers of grains per ear, and the number of ears within a specific area (Gegas et al., 2010). These are in turn influenced by other traits, such as accumulation and transport of photosynthetic products, flag leaf size, plant height, biomass, and the rate of plant development across its lifecycle (most commonly determined by measurement of flowering time). Additionally, the postharvest processing of wheat grain for subsequent food use mean that traits that affect milling performance, such as the shape, size, density, and uniformity of the grain, are important for flour yield. In addition to yield, there are several grain quality characters that are important in determining end use, and thus the monetary value of the grain. In the United Kingdom, wheat cultivars are classified into four quality groups by the National Association of British and Irish Millers (Nabim, http://www.nabim.org/), based on grain protein content, HFN (a measure of α‐amylase enzymatic activity in sprout‐damaged grain) and test weight (a measure of grain density, determined by the weight of a set volume of grain). Nabim Group 1 and Group 2 cultivars are high protein, hard grained cultivars suitable for bread making and attract a price premium. Group 3 represents cultivars with lower protein (∼11%) and soft milling characteristics due to their soft grain phenotype and are typically used for biscuit making. Group 4 cultivars have low protein content, can be either hard or soft grained, and are used predominantly for animal feed (UK Flour Millers, 2017).

**Core Ideas**

- An association mapping panel of 150 genotyped U.K. winter wheats was assembled.
- Data for four grain quality traits was sourced from cultivar registration records.
- Genome‐wide association studies (GWAS) identified at least five QTL for each trait.
- A major GWAS hit for Hagberg falling number was identified on chromosome 7A.
- A third of yield GWAS hits co‐located with genetic loci under breeder selection.

Protein content in wheat determines how flour performs during baking and is normally measured as percent of dry matter using near infrared spectrometry (NIR). For bread making (Nabim Groups 1–2), grain protein content values of ≥13% are required, whereas for biscuit making (Group 3), grain protein content levels are typically 11–11.5% (UK Flour Millers, 2017). In addition, protein content represents an important factor for the nutritional value of the grain or flour: annual wheat production is estimated to be ∼730 Tg (FAO, 2016), which translates to ∼73 Tg of protein for human and animal consumption. Protein is predominantly present in the form of grain storage proteins, including the high molecular weight glutenin subunits (HMW‐GS), low molecular weight glutenin subunits (LMW‐GS), and the gliadins. Grain quality is determined by the quantity and quality of the storage proteins present, as well as the ratio of glutenin to gliadin (Jouanin et al., 2018).

The HFN (Hagberg, 1960) is an internationally standardized method to assesses starch properties in wheat flour. The test measures the time in seconds a stirrer that is placed in a test tube of heated coarse meal‐water mix takes to fall down through the mixture, thus determining the effects of α‐amylase enzymes on starch properties within the mixture (Mohler et al., 2014). The HFN is principally determined by two factors: preharvest sprouting (PHS), a loss of dormancy resulting in premature germination of the grain while still on the parent plant, and late‐maturity amylase, the untimely synthesis of high levels of high isoelectric point isozymes of α‐amylase during the latter stages of grain development, without sprouting. In both cases, α‐amylase activity during the later stages of grain development lead to starch breakdown and reduced grain quality (Mares & Mrva, 2008), resulting in lower HFN values due to reduced viscosity of the mixtures used to assess HFN. In the United Kingdom, grain suitable for bread making must have a HFN ≥250 s (UK Flour Millers, 2017). Grain which doesn't meet this threshold result in breads with lower volume and a compact, sticky crumb structure (Gale & Lenton, 1987).

Test weight, also known as specific weight, is a measurement of the weight of a known volume of grain and is an important indicator of quality. Low test weight can indicate poorly filled or misshapen grains, and/or high moisture content. Wheat with a low test weight generally results in low flour extraction rates. For bread making quality, test weights of more than 76 kg ha^−1^ are generally required (UK Flour Millers, 2017). Relatively few studies reporting QTL for test weight have been published (e.g., Narasimhamoorthy et al., 2006), one of the most recent of which identified eight loci were identified on chromosomes 1D, 2A, 2B, 2D, 3B, 3D, 4D, and 7A (Cabral et al., 2018). However, test weight is largely determined by grain weight, shape and volume, and so can be broken down into its constitutive components and investigated as separate yield components (e.g., Corsi et al., 2021).

Previous genome‐wide association study (GWAS) analysis in U.K. wheat have used the available historical yield data to identify QTLs controlling grain yield, identifying QTL located on chromosomes 1A and 5A (Sharma et al., 2021). Here, the aim of our study was to use historical grain quality and yield datasets to identify genetic loci controlling these traits. To this end, a panel of 150 elite U.K. winter wheat cultivars genotyped with a 90,000‐feature single nucleotide polymorphism (SNP) genotyping array was used to perform GWAS for the three grain quality traits measured during wheat registration in the United Kingdom: grain protein content, HFN, and test weight. Although our panel overlaps with to some extent with that recently used by Sharma et al. (2021) to undertake GWAS on grain yield, we also include yield in our analysis here, principally to help any direct comparisons with marker‐trait association made for our analysis of grain protein content, HFN, and test weight. We discuss the genetic loci identified in the context of previously published QTL.

## 2 MATERIALS AND METHODS

### 2.1 Wheat germplasm

An association mapping panel of 150 U.K. winter wheat cultivars was assembled, consisting of lines released between 1965 and 2004, and that had undergone at least 3 yr of phenotypic evaluation under Home Grown Cereals Authority (HGCA; Since termed the ‘Agriculture and Horticulture Development Board’) National List trials between 1975 and 2007 (Supplemental Table S1). Seed of the cultivars was obtained from seed banks (John Innes Centre, UK; IPK Genebank, Germany; USDA National Small Grains Collection, USA), or from a reference seed collection held by NIAB on behalf of Defra, with the written permission of the relevant breeding companies.

### 2.2 Historical phenotypic data, estimates of heritability, and trait correlations

Historical phenotypic data recorded during HGCA Recommended List trials for grain protein content (measured as percent dry matter), HFN (measured in seconds), and test weight (the weight in grams of seed that can be packed into a 1‐L standard container) from HGCA trials between 1975 and 2007 were sourced, with permission, from HGCA Recommended List trial data (Table 1). Historical phenotyping followed standard HGCA National List protocols (http://www.hgca.com/). Estimates of cultivar effect for each trait were obtained from historical experimental data originating from UK National List and Recommended List trials (1950–2004). The variance structure of the phenotypic data in a mixed effects model was estimated using REML (Genstat Version v.11, VSN International). We estimated best linear unbiased estimates using the models summarized in Supplemental Table S2. Heritability (*h* ^2^), more strictly defined as repeatability, was estimated from the random model compromising only main effects, the denominator of the error term being estimated from the ratio of stratum variance to variance component for cultivar. Phenotypic correlations were investigated using R‐package “Hmisc” and the correlation values highlighted in Microsoft Excel 2016 using conditional formatting. Where possible, Nabim group (1, 2, 3, or 4) classification was sourced from Agriculture and Horticulture Development Board Recommended List data, for Year 2004 onwards, available online at https://ahdb.org.uk/knowledge‐library/recommended‐lists‐archive. Prior to 2004, data was sourced from nondigitized NIAB Pocket Handbooks, and for those cultivars which predated the introduction of formal Nabim group classification in the Recommended List datasets, group was estimated based on the available quality data.

**TABLE 1:** The volume and structure of historical winter wheat trials data

					**Averages**				
** Trait**	**No. samples**	**No. years**	**No. vars**	**No. trials**	**Trial size (no. vars.)**	**Trials per year**	**Replications per var**.**^a^**	**Var. years in trial**	**Var. replications per year**
Yield	74,332	32	1,219	1,472	50.5	46.0	61.0	2.2	22.0
HFN	21,458	31	1,324	586	36.6	18.9	16.2	2.0	8.1
Protein	17,097	31	1,145	691	24.7	22.3	14.9	1.8	8.4
TW	18,452	31	1,211	546	33.8	17.6	15.2	1.7	9.1

*Note*. HFN, Hagberg falling number; Protein, protein content; TW, test weight; Var., variety.

^a^ The average number of estimations of the trait for a cultivar in the database.

### 2.3 Genotypic data and principal component analysis

Genotypic data for the 150 cultivars, generated using the 90,000 feature Illumina SNP array (Wang et al., 2014), was sourced from https://www.niab.com/pages/id/326/Resources. Principal component analysis (PCA) was undertaken using 16,801 genetically mapped markers as well as a set of 463 skimmed markers parsed using TASSEL (Bradbury et al., 2007) and further thinned to a minimum distance between markers of 10 cM. Information on the presence or absence of the 1B/1R wheat/rye chromosomal translocation (which has been widely used in wheat breeding programs as a source of beneficial traits such as disease resistance; Rabinovich, 1998) in the varietal panel was overlaid onto PCA plots.

### 2.4 GWAS, bioinformatics, and power analysis

The SNPs were parsed based on minor allele frequency (MAF) ≥0.05 and *R^2^* ≥ .2. The GWAS was undertaken using a mixed linear model to fit a linear mixed model with cultivars treated as random and SNPs effects as fixed. To account for population substructure in the association mapping panel, centered identity by state (IBS) based kinship was used. For the kinship matrix generation, a subset of markers was selected based on linkage disequilibrium (LD) implemented in the R‐package SNPRelate (Zheng et al., 2012). The following equation was fitted in the mixed linear model: y=Xβ+Zu+e, where **y** is a vector representing traits values; β is the fixed effect of the SNP under test; **u** refers to a vector of random additive genetic effects for the 150 lines; **e** is the vector of error terms; and **X** and **Z** represents known design matrices. More details are provided by Yu et al. (2006). Analysis was performed using options optimum compression level and P3D variance component estimation as implemented using the mixed linear model (MLM) method within the software TASSEL version 5.2.54 (Bradbury et al., 2007). Additionally, MLM method with both population structure and kinship relationship (MLM‐PCA‐K) was employed. In the Manhattan plots, SNPs are arranged in genetic map order (Wang et al., 2014), with unmapped markers excluded from the plots. An arbitrary significance threshold of −log_10_ *P *= 3 was used to report marker‐trait associations. This threshold was supported by our power calculations, which estimated a false positive rate of 0.001 (i.e., −log_10 _= 3). Significant markers within a conservatively defined 15‐cM interval of each other were treated as identifying the same QTL. For each trait, the percentage of variation explained (PVE) was determined in two ways: (a) via the outputs of the software TASSEL; and (b) specifically just for the QTL detected by GWAS, calculated in R (R Core Team, https://www.rproject.org/contributors.html) using the regression lm function without population structure adjustments. The positions of selected SNPs on the wheat reference genome of ‘Chinese Spring’ (genome assembly RefSeq v1.0; IWGSC, 2018) were identified using the Triticeae Toolbox database via https://triticeaetoolbox.org/wheat/, which anchors SNPs based on nucleotide basic local alignment search tool (BLASTn) homology. Where an SNP was anchored to a different homoeologous chromosome to that identified in the genetic map (Gardner et al., 2016), physical map location was based on manual BLASTn analysis using Ensembl Plants (Yates et al., 2020). For cross‐comparison of our GWAS hits with published QTL, other genetic marker types were anchored in a similar way, after extraction of available sequence data from GrainGenes (https://wheat.pw.usda.gov/GG3/) or in the case of Diversity Array Technology (DArT) markers, from information downloaded from https://www.diversityarrays.com/technology‐and‐resources/sequences/. Power calculations were performed using the custom R‐scripts detailed in Supplemental Text S1, which implemented the power calculation functions described by Wang and Xu (2019). For this, the kinship matrix was decomposed into eigenvalues and their corresponding eigenvectors. The eigenvalues were subsequently used to compute power using functions provided in the power calculations scripts published by Wang and Xu (2019). Power was calculated for QTL heritability values ranging from .01 to .4 and for linkage disequilibrium ranging from *R* ^2 ^= .011 to *R* ^2 ^= 1.

### 2.5 Associations with Nabim classifications

To test whether the classification of cultivars into Nabim quality groups accounted for any population structure, we used multiple regression of cultivar loadings of each of the first two principal components onto the Nabim classifications, with chromosome 1B/1R translocation, protein content, test weight, HFN, and grain hardness traits included as additional covariates. The importance of the Nabim classification was estimated by statistical significance in analysis of variance (ANOVA), with the factor for Nabim fitted both before and after the other covariates.

## 3 RESULTS

### 3.1 Analysis of phenotypic and genotypic data

**FIGURE 1:**
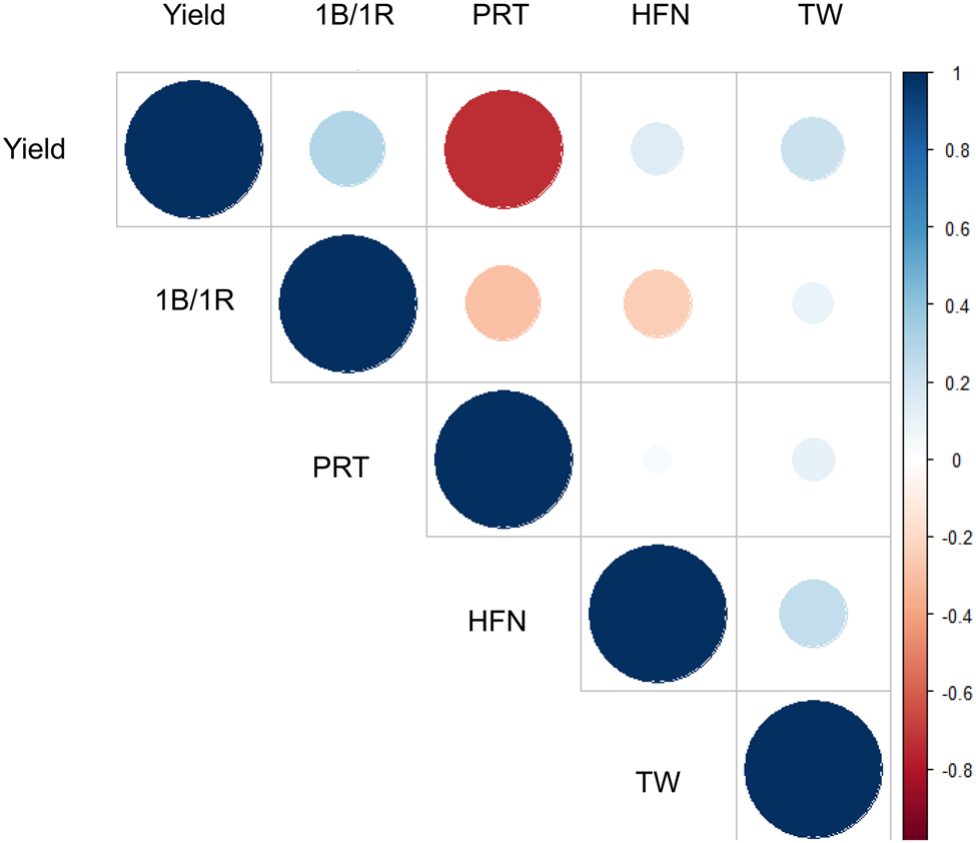
Pearson correlation between grain yield (Yield), the presence or absence of the wheat/rye chromosome 1B/1R translocation, grain protein content (PRT), Hagberg falling number (HFN), and test weight (TW)

Phenotypic data for protein content, HFN, and test weight were extracted from historical HGCA National List datasets. Summary data for each trait (including the number of samples, years, cultivars, and trials, as well as mean values) are listed in Table 1. Estimation of the variance structure of the phenotypic data is shown in Table 2. Random main effects were fitted for years, sites within years, management regimes, and cultivars. Residual variance was estimated from a model fitting random main effects for years, sites within years, management regimes, and cultivars. Average replication per cultivar was taken from the table of stratum variances. Estimates of *h* ^2^ for all traits was ≥.86, with grain yield showing the highest value (.95), followed by test weight (.90), HFN (.89), and protein content (.86). These high values are inevitable given the high average numbers of years of testing, locations, replication number, and high genetic variances. Explicitly for yield, in addition to far higher replication of locations and years in comparison to almost all contemporary experiments, the genetic variation is substantially inflated by inclusion of old and modern cultivars, spanning 40 years, during which period yield was increased substantially by genetics alone. Best linear unbiased estimates were estimated for all four traits and are listed in Supplemental Table S1. Traits showed broadly normal distributions, although yield was notable skewed towards higher values (Figure 1). Analysis of correlation between our three target traits, as well yield, found showed strong negative correlation between yield and protein content (*P *> .0001), as well as significant negative correlations between the 1B/1R translocation and protein (*P =* .0003) and HFN (*P *= .0183) (Figure 1; Supplemental Table S3). Significant positive correlations were found between the 1B/1R translocation and yield (*P *= .0004), and between test weight and both HFN (*P *= .0002) and yield (*P *= .0124).

**TABLE 2:** Variance components and trait heritability

	**Variance components**	**Mean square**		**(VarMS − ResMS)/V_g_**	**V_g_**	**ResMS/ n_v_**	**V_g_/(V_g_ + V_e_)**
**Trait**	**V_g_**	**Cultivar**	**Residual**	**n_v_**		**V_e_**	**Trait *h* ^2^ using all data**
Yield	0.393	10.265	0.400	24.89	0.393	0.020	0.950
Protein	0.226	2.017	0.287	7.67	0.226	0.037	0.858
HFN	1591	13,287	1,437	7.45	1591	192.89	0.892
TW	2.580	20.150	2.020	7.03	2.580	0.287	0.899

*Note*. *h* ^2^, heritability; HFN, Hagberg falling number; nv, average replication per cultivar; Protein, protein content; ResMS, residual mean square; TW, test weight VarMS, varieties mean square; Vg, genetic variance; Ve, environmental variance.

### 3.2 Population structure and power calculations

**FIGURE 2:**
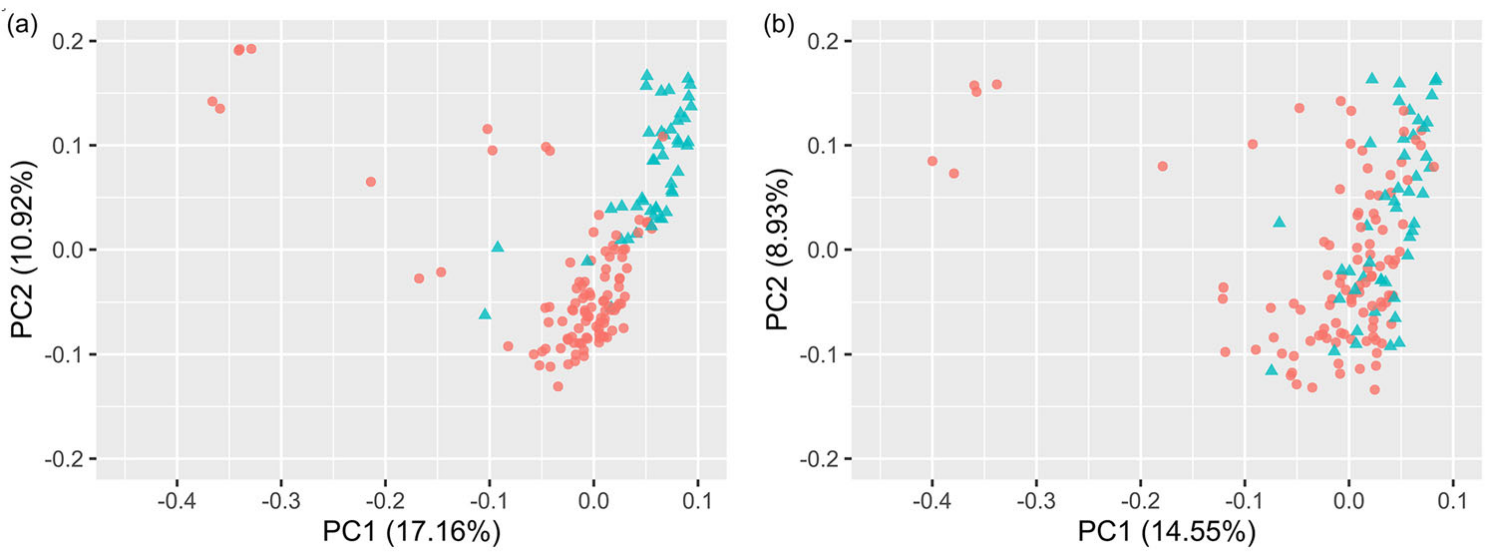
Population subsubstructure in the association mapping panel of 150 accessions, based on (a) all markers (*n *= 16,801), and (b) skimmed markers (*n *= 463; linkage disequilibrium threshold *R^2^* = .2). Markers with a minor allele frequency >0.05 were used for both analyses. The presence or absence of the chromosome 1B/1R wheat/rye chromosomal substitution is indicated by red circles and blue triangles, respectively. PC, principal component

Genetic population substructure was investigated using a matrix of similarity between cultivars for PCA (Figure 2). The PCA using 16,801 polymorphic genetically mapped markers revealed a clear division in the first principal component. Overlaying data for the presence or absence of the 1B/1R wheat/rye chromosomal translocation indicated that the division based on Principal Component 2 was largely due to this chromosomal rearrangement (Figure 2). While PCA analysis after removal of one of each pair of highly correlated (*R* ^2 ^> .98) markers removed much of this distinction (Figure 2), the presence of further genetic substructure indicated statistical correction would likely be necessary for genome‐wide association studies. To determine whether classification of cultivars into the four Nabim end‐use grain quality groups could explain significant amounts of PCA space, we undertook analysis of variance. Nabim was found to explain a modest amount of the variation for each of the two PCA components (16% of PCA1, *p =* .0017**; 8% of PCA2, *p =* .0306*). However, Nabim group is a man‐made ‘trait’, and is determined by a number of underlying quality traits. When such component traits (1B/1R translocation, protein content, test weight, HFN, grain hardness) were fitted before Nabim classification, then Nabim classification no longer identified significant variation in either PCA1 or PCA2 space.

To estimate the power of the experimental design to detect QTL, we undertook power analyses. These found that despite low number of accessions of this study (*n =* 150) this association mapping panel was predicted to be able to detect QTL of reasonable effects (Supplemental Table S4). For example, the analysis predicts us to be able to identify QTL with a heritability of ≥20% with high power of >80%, with power falling to 40% for a SNP in LD of *R* ^2^ ≥ .5 with a given QTL. With the relatively high density of markers in this panel, it is likely that most QTL will be in high LD with one or more markers (data not shown)—at least on the A and B subgenomes which have much higher marker density than the D subgenome (6,188, 8,488, and 2,125 genetically mapped markers, respectively).

### 3.3 Genome‐wide association studies

**FIGURE 3:**
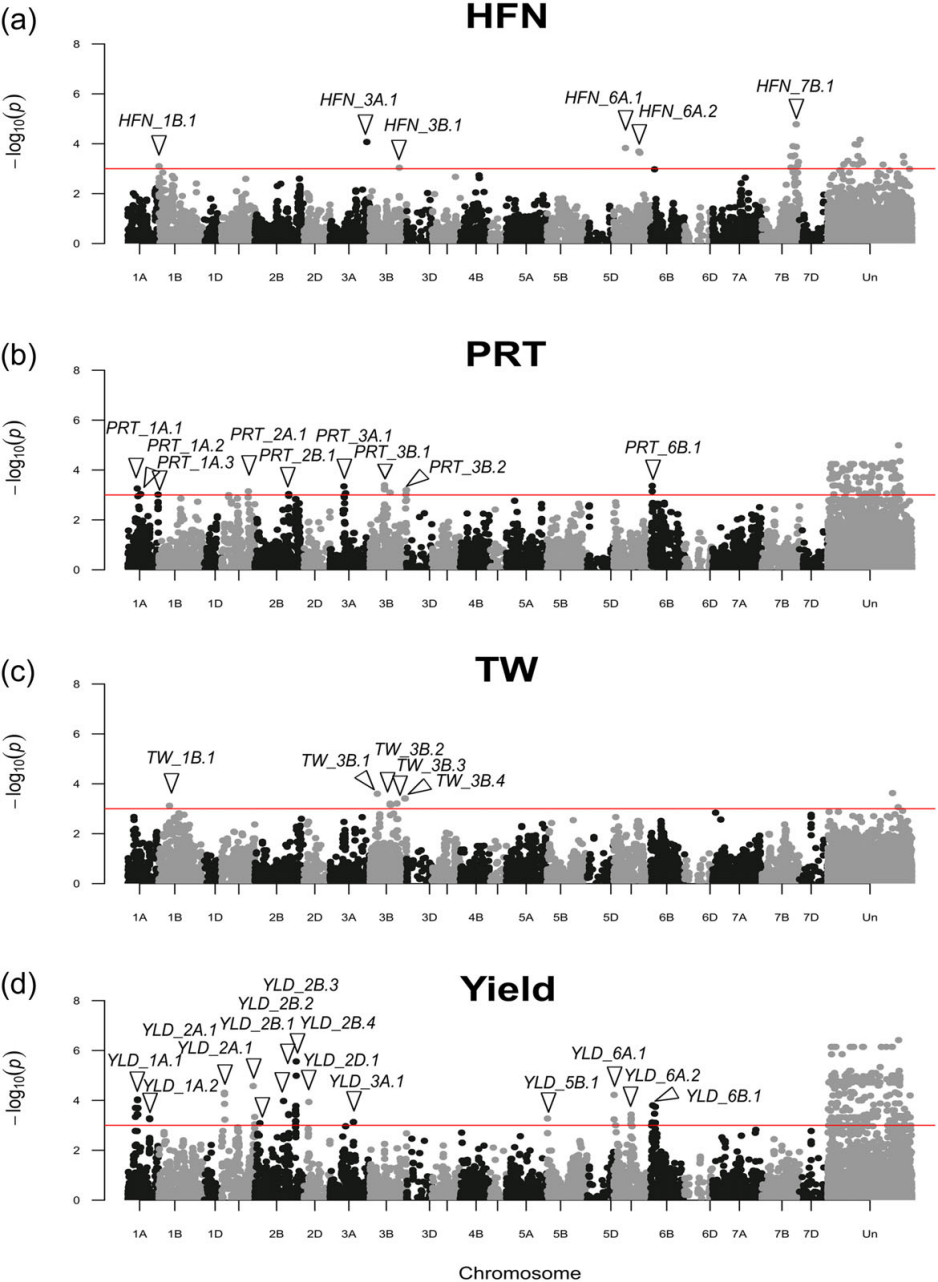
Manhattan plots of genome‐wide association studies for Hagberg falling number (HFN; a), protein content (PRT; b), test weight (TW; c), and grain yield (Yield; d). Markers are shown in genetic map order, according to the genetic map published by Wang et al. (2014). Unmapped markers are not shown here but are listed in Supplemental Table S5. The significance threshold (−log_10_ *P* = 3) is indicated by the horizontal red line

**FIGURE 4:**
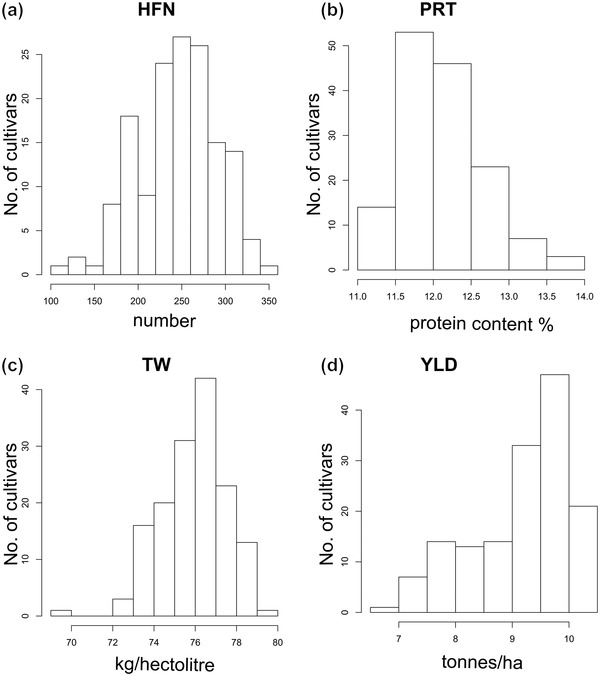
Histogram of Hagberg falling number (HFN; a), protein content (PRT; b), test weight (TW; c), grain yield (YLD; d) in the association mapping panel

After removal of markers with MAF ≤0.05, the final data‐matrix for GWAS consisted of 150 cultivars and 20,921 SNPs (including 5,515 unmapped SNPs). After accounting for population substructure using a Kinship matrix, the results of GWAS identified significant (−log_10_ *P *>3) marker‐trait associations for all four of the phenotypes investigated. The GWAS results are displayed as Manhattan plots in Figure 3, histograms of the phenotypic trait expression are shown in Figure 4, and the genomic hits are summarized in Table 3, with the GWAS outputs for all markers used listed in Supplemental Table S5. The adoption of a significance threshold of −log_10_ *P =* 3 was arbitrary, but an excess of low *P*‐values for all four traits is apparent from the Q‐Q plots and histograms of *P*‐values shown in Supplemental Figure S1. For HFN, 39 significant genetic markers with −log_10_ *P*‐values ranging from 3.10 to 3.00 were identified, which when modelled together explained 49.0% of the phenotypic variance. Of these, 24 were genetically mapped and defined six genetic loci on chromosomes 1B (locus *HFN_1B.1*), 3A (*HFN_3A.1*), 3B (*HFN_3B.1*), 6A (*HFN_6A.1*, *HFN_6A.2*), and 7B (*HFN_7B.1*). The GWAS for protein content identified 151 significant marker‐trait associations (−log_10_ *P* from 4.99 to 3.01) with the 41 genetically mapped markers, coalescing into nine genetic locations on chromosomes 1A (locus *PRT_1A.1, PRT_1A.2, PRT_1A.3*), 2A (*PRT_2A.1*), 2B (*PRT_2B.1*), 3A (*PRT_3A.1*), 3B (*PRT_3B.1, PRT_3B.2*, *PRT_3B.3*), and 6B (*PRT_6B.1*). Together, these QTL were modelled to explain 48.9% of the phenotypic variance. The GWAS for test weight identified 39 significant marker‐trait associations (−log_10_ *P* from 3.63 to 3.06), defining five genetic loci located on chromosomes 1B (*SW_1B.1*) and 3B (*SW_3B.1, SW_3B.2, SW_3B.3, SW_3B.4*), plus two unmapped markers. When modelled together, these QTL explained 26.3% the phenotypic variance. Finally, GWAS for yield identified 533 significant SNPs (−log_10_ *P* from 6.42 to 3.00). Of these, 351 were unmapped with the remaining 182 genetically mapped SNPs identifying 14 genetic loci on chromosomes 1A (*YLD_1A.1, YLD_1A.2*), 2A (*YLD_2A.1, YLD_2A.2*), 2B (*YLD_2B.1, YLD_2B.2, YLD_2B.3, YLD_2B.4*), 2D (*YLD_2D.1*), 3A (*YLD_3A.1*), 5B (*YLD_5B.1*), 6A (*YLD_6A.1, YLD_6A.2*), and 6B (*YLD_6B.1*) which when modelled together explained 68.1% of the phenotypic variance. We also undertook GWAS after adjusting for population structure using kinship + PCA (Supplemental Table S6). Overall, the results remained much the same, although some hits were lost (as indicated in Table 6) and some were gained.

**TABLE 3:** Summary of the quantitative trait loci (QTL) identified by genome wide association studies (GWAS) for Hagberg falling number (HFN), protein content (PRT), test weight (TW), and grain yield (YLD)

**Trait**	**QTL**	**Chr**	**Peak SNP**	**cM**	**bp**	**−log_10_ *P***	**Marker R^2^** **^a^**	**Effect** ^b^	**Allele Freq**
HFN	*HFN_1B.1*	1B	Jagger_c5878_119	0	1,254,498	3.10	0.079	28.42	0.58
HFN	*HFN_3A.1* ^c^	3A	RAC875_c99055_69	283.735979	728,322,627	4.08	0.114	−30.65	0.47
HFN	*HFN_3B.1*	3B	BS00099738_51	227.3745367	759,168,681	3.04	0.077	−31.32	0.73
HFN	*HFN_6A.1* ^c^	6A	Kukri_c29110_360	88.77758706	Un	3.83	0.103	−31.56	0.61
HFN	*HFN_6A.2* ^c^	6A	BS00082104_51	192.8384162	581,841,966	3.69	0.098	32.95	0.38
HFN	*HFN_7B.1* ^c^	7B	RAC875_c525_202	261.6310052	750,082,927	4.78	0.134	−44.19	0.19
PRT	*PRT_1A.1*	1A	BS00086680_51	71.0976257	281,657,026	3.26	0.087	0.39	0.52
PRT	*PRT_1A.2*	1A	Excalibur_c13489_867	96.6018344	452,020,371	3.03	0.079	−0.36	0.38
PRT	*PRT_1A.3*	1A	RAC875_c5882_307	231.6429733	589,054,742	3.01	0.079	−0.33	0.34
PRT	*PRT_2A.1*	2A	RAC875_rep_c69619_78	207.5522799	734,352,032	3.15	0.083	−0.33	0.56
PRT	*PRT_2B.1*	2B	BS00046165_51	260.7730538	697,510,384	3.04	0.080	0.44	0.19
PRT	*PRT_3A.1* ^c^	3A	wsnp_Ku_c30545_40369365	107.2210875	363,458,708	3.34	0.090	−0.35	0.52
PRT	*PRT_3B.1* ^c^	3B	wsnp_Ex_c20652_29734133	112.6476302	292,024,034	3.39	0.091	−0.44	0.79
PRT	*PRT_3B.2*	3B	RAC875_c58159_989	155.9566423	564,248,743	3.10	0.081	−0.46	0.83
PRT	*PRT_3B.3* ^c^	3B	GENE_1618_780	279.0622821	820,894,420	3.02	0.079	−0.33	0.52
PRT	*PRT_6B.1* ^c^	6B	BS00009795_51	12.20934913	4,876,473	3.36	0.090	‐0.35	0.44
TW	*TW_1B.1* ^c^	1B	RAC875_rep_c95069_54	81.49097047	336,647,904	3.12	0.080	−1.24	0.21
TW	*TW_3B.1* ^c^	3B	GENE_1771_541	57.06175117	32,458,901	3.60	0.095	1.20	0.36
TW	*TW_3B.2* ^c^	3B	RAC875_c58159_989	155.9566423	571,753,368	3.20	0.083	−1.43	0.86
TW	*TW_3B.3* ^c^	3B	Excalibur_c33274_498	207.7302565	738,752,902	3.21	0.083	2.04	0.05
TW	*TW_3B*.	3B	BS00073480_51	269.7509427	812,721,677	3.41	0.089	−1.91	0.91
YLD	*YLD_1A.1* ^c^	1A	RAC875_rep_c105092_114	72.6102779	304,040,711	4.03	0.109	0.62	0.50
YLD	*YLD_1A.2* ^c^	1A	wsnp_CAP8_c4785_2322876	166.3032581	544,054,745	3.28	0.085	0.56	0.79
YLD	*YLD_2A.1* ^c^	2A	RAC875_c48625_182	22.63785838	18,636,671	4.30	0.118	0.57	0.53
YLD	*YLD_2A.2* ^c^	2A	RAC875_c16993_839	245.2533379	774,815,015	4.57	0.127	−0.73	0.38
YLD	*YLD_2B.1*	2B	BS00002660_51	38.40853424	16,025,160	3.09	0.079	0.50	0.57
YLD	*YLD_2B.2* ^c^	2B	BS00091099_51	221.2304675	578,601,402	3.97	0.107	−0.55	0.54
YLD	*YLD_2B.3* ^c^	2B	BS00046165_51	260.7730538	697,510,384	3.45	0.090	−0.70	0.19
YLD	*YLD_2B.4* ^c^	2B	Kukri_c34553_188	319.3174469	766,234,466	5.56	0.161	−1.00	0.17
YLD	*YLD_2D.1* ^c^	2D	wsnp_Ex_c1668_3169623	35.64284331	Un	3.94	0.106	0.59	0.70
YLD	*YLD_3A.1*	3A	wsnp_Ex_c8884_14841846	179.8202073	625,239,797	3.13	0.08027	−0.51	0.73
YLD	*YLD_5B.1* ^c^	5B	CAP8_rep_c5825_165	3.286161131	15,054,913	3.27	0.0847	0.5	0.4
YLD	*YLD_6A.1* ^c^	6A	BS00082812_51	0.502519793	639,383	4.22	0.11534	0.73	0.78
YLD	*YLD_6A.2* ^c^	6A	wsnp_Ku_c3354_6228393	132.9525631	430,933,984	3.43	0.09203	−0.71	0.24
YLD	*YLD_6B.1* ^c^	6B	Kukri_c21405_2131	15.73078008	166,814	3.8	0.10161	−0.73	0.19

*Note*. The most significant marker at each QTL is listed. Genetic (Gardner et al., 2016) and physical (IWGSC, 2018) map positions for peak single nucleotide polymorphisms (SNPs) at each QTL are indicated. Where SNPs were anchored to physical map regions currently not allocated to a chromosome designation in the wheat reference genome assembly, the bp position is recorded as unknown (Un). SNP effect at each QTL were determined via two methods (defined below).

^a^ SNP effects determined via the outputs of the software TASSELL.

^b^ SNP effects determined via modeling the effects considering just those QTL identified for a specific trait.

^c^ GWAS hits identified using both kinship and kinship+PCA to correct for population structure. The remaining hits were identified using kinship correction only.

## 4 DISCUSSION

### 4.1 Exploiting historical phenotypic data

Recent advances in molecular marker technologies in Triticeae species, such as Diversity Array Technology (DArT) markers, SNP arrays (e.g., Close et al., 2009; Wang et al., 2014), and Kompetitive Allele Specific Polymerase chain reaction (KASP) assays (e.g., Cockram et al., 2012) have meant that phenotyping capacity and capability is now widely recognized as having replaced genotyping as the major bottleneck for genetic studies (Furbank & Tester, 2011). A central feature of this study is that the yield and quality phenotypes investigated were obtained from historical databases. Such data have been accumulated in many countries as a normal part of cultivar testing, either by breeders or testing authorities under the internationally recognized protocols established by the Union for the Protection of plant Varieties (UPOV) and are increasingly used in association mapping studies (e.g., Cockram et al., 2010; Wang et al., 2012) and the development of molecular approaches for use in cultivar testing and registration (Cockram et al., 2009, 2012; Jones, Norris, Smith et al., 2013; Jones, Norris, Cockram, & Lee, 2013; Saccomanno et al., 2020). Historic analyses of trends in cultivars over time have used regression models to infer year, site, and treatment effects to allow the genetic potential of cultivars to be compared, even when the two cultivars were never in the same field trial (Mackay et al., 2011; Silvey, 1978, 1981, 1986). While year‐to‐year variability (which includes the joint effect of differences in management, climate, and noise over the years) is accounted for in our models (Supplemental Table S2), here we focus on genetic effects, as used in our GWAS analyses. A key advantage of using historical phenotypic data collected as part of cultivar registration is that the resulting panel consists of elite material. This is of benefit as QTL identified are more likely to be of direct relevance to ongoing breeding programs. Additionally, elite panels are more useful than those consisting of exotic or diverse materials for traits strongly influenced by biotic or abiotic stresses and differences in maturity (and therefore limiting meaningful phenotypic data collection; Kulwal et al., 2012).

### 4.2 Characteristics of the association mapping panel used

Association mapping panels consisting of collections of cultivars, landraces, or breeder's lines are likely to have hidden kinship and population structures (White et al., 2008). Thus, kinship and population structure are likely to be strong predictors of many traits (e.g., Cockram et al., 2008). In this study, major genetic stratification was found due to the presence of the wheat/rye chromosome 1B/1R translocation. Parsing marker sets on MAF and *R* ^2^ removed much of this substructure, with residual effects of relationships among cultivars removed though incorporation of a relationship matrix in the linear mixed model. The dataset analyzed encompasses all the trait data available to us. While a larger dataset would have been preferable, our power calculations taken in conjunction with the Q‐Q plots of −log_10_ *P* and histograms of *P*‐values demonstrate that despite the relatively small sample size of *n *= 150, a substantial proportion of our significant results are genuine. The data we use is ‘the best of the best’ in the sense that the lines have already been selected by the breeders, leading to potential selection bias in QTL discovery. We do not believe this is a problem for two reasons. First, it should be accounted for by inclusion of the genomic relationship matrix in the GWAS. Second, the heritabilities of our line means are very high, so the opportunity for selection of cultivars to bias the results or QTL segregating among those cultivars is low. Within this study, there is potential for selection bias arising from selection of lines being advanced from assessment Year 1 to Year 2 and so on. This may result in some “regression to the mean” of cultivar estimates. Laidig et al. (2014) discussed this source of bias in relation to estimating genetic and agronomic trends, finding only a slight bias in trend estimation. However, we are not aware of discussion of the consequences of varietal selection bias on the effect of QTL estimation in GWAS. Our expectation is it will be small compared with the effect of selection bias resulting from the imposition of a significance threshold on QTL detection (the ‘Beavis effect’; Beavis, 1994), since both increasing and decreasing alleles for QTL of small effect will be approximately randomized across lines, and the bias for QTL of large effect will be lower whatever their distribution across cultivars. However, we are not aware that this potential source of bias, which will affect almost all GWAS studies in crops, has been studied and merits further investigation.

### 4.3 QTL detection, phenotypic correlations, and comparison with previous studies

While a relatively limited number of studies in investigating the genetics of HFN have been undertaken to date, HFN QTL have been detected on numerous wheat chromosomes (e.g., Börner et al., 2018; Fofana et al., 2009; Gooding et al., 2012; Guo et al., 2020; Kunert et al., 2007; Li et al., 2020; Martinez et al., 2018; Mohler et al., 2014; Tang et al., 2017; Zanetti et al., 2020; Zhang et al., 2014). Based on the interconnection between HFN, PHS, and late‐maturity amylase phenotypes, these traits likely share QTL in common (Kulwal et al., 2010). The PHS QTL have been mapped to all 21 wheat chromosomes (reviewed by Kulwal et al., 2010), and the most commonly reported loci are on the Group 2 chromosomes (2B, 2D), the Group 3 chromosomes (3A, 3B, 3D, associated with the wheat *VIVIPAROUS1* gene *TaVP1*, and the red kernel color *R* genes), as well as *Pre‐harvest sprouting 1* (*Phs1*, synonym: *Phs‐A1*) on the long arm of chromosome 4A (Flintham, 2000; Groos et al., 2002; Jaiswal et al., 2012; Kulwal et al., 2012; Mares et al., 2005; Mori et al., 2005; Munkvold et al., 2009; Shorinola et al., 2016). Similarly, QTL for late‐maturity α‐amylase have been found on chromosomes 3B, 6B, and 7B (Emebiri et al., 2010; Mrva & Mares, 2001; 2002). In our study, six genetic loci controlling HFN were identified, the majority of which were located within genetic intervals spanning previously identified QTL related to the HFN phenotype (Supplemental Table S6). The most significant locus we identified was on the long arm of chromosome 7B (located at 725–750 Mbp; peak at 750 Mbp), for which favorable alleles increased HFN by 44 s. The QTL for HFN have previously been identified around this region in at least four biparental populations, with high falling number alleles contributed by the wheat cultivars Rubens (QTL peak at 709 Mbp; Börner et al., 2018), Dream (no SNP sequence data available), W332‐84 (744 Mbp), Format (744 Mbp; Mohler et al., 2014), and the winter spelt cultivar Oberkulmer (741 Mbp; Zanetti et al., 2020), resulting in an increase of between 18 and 75 s, depending on the population and test environment. Our 7B locus is in a similar genomic region to a QTL for high‐isoelectric point α‐amylase content which is used as a measure of late maturity α‐amylase activity (Mrva & Mares, 2001; Emebiri et al., 2010) (Supplemental Table S6). As HFN and α‐amylase activity are genetically correlated and inversely related, it suggests that the same locus likely underlies the 7B QTL for these traits. Our chromosome 1B HFN locus (located at 1.254 Mbp) was associated with the 1BL/1RS wheat/rye chromosomal translocation, for which analysis of exome capture sequence coverage and nonreference allele frequency relative to the wheat genome assembly for Chinese Spring found to be located on chromosome 1B between 0 and 220 Mbp (Scott et al., 2021). The inclusion of the 1B/1R translocation as a covariate in the GWAS analysis resulted in the markers at the 1B HFN locus becoming nonsignificant (−log_10_ *P *= 2.01), consistent with the HFN locus *HFN_1B.1* being located on the translocation itself (although this result does not rule out *HFN_1B.1* being in high linkage disequilibrium with the translocation). The indication that the 1B HFN locus may present on the translocation is in agreement with previous studies (Mohler et al., 2014; Tang et al., 2017) and reflects the strong negative correlation between HFN and the presence of the 1B/1R translocation identified in our association mapping panel (Figure 1). Therefore, in our analysis, the absence of the 1B/1R translocation was predicted to result in an HFN increase of 28 s. Previous analysis of near isogenic lines has shown that gibberellic insensitive semidwarf lines that lack the 1B/1R translocation require a cool temperature shock during grain filling for the expression of late maturity α‐amylase activity while the presence of the translocation results in constitutive expression (Mrva et al., 2008). In both classes of response, late maturity α‐amylase activity is due to the synthesis of high‐isoelectric point α‐amylase encoded by genes at the *α‐Amylase1* (*α‐Amy1*) locus at 554–564 Mbp on the long arm of chromosome 6A, at which at least three genes are present (RefSeq v1.1 gene models *TraesCS6A02G319300*, *TraesCS6A02G334100*, *TraesCS6A02G334200*; Ju et al., 2019). Interestingly, we also identified a QTL for HFN on chromosome 6A at 581 Mbp (second only to the 7B locus in its effect on HFN), indicating this GWAS hit could be due to allelic variation at the *Amy1* locus. Indeed, a QTL at a similar location on chromosome 6A has been reported in German winter wheat (Mohler et al., 2014; Supplemental Table S6). Lastly, while our HFN loci on chromosomes 3A (728 Mbp) and 3B (759 Mbp) were identified at approximately homoeologous locations on the long arms of the Group 3 chromosomes, they were not located close to *VP‐1A* (660 Mbp) or *VP‐1B* (693 Mbp). Neither did our 3B QTL appear to correspond to the QTL *QFn.crc‐3B* previously reported to be located between markers *barc77* and *wmc307* (3B: 78–430 Mbp; Fofana et al., 2009). However, they were located relatively close to the wheat *MYB* gene *TaMYB10‐A1* located on chromosome 3A at 704 Mbp, and its homologue *TaMYB10‐B1* on 3B at 571 Mbp. *TaMYB‐A1* has previously been identified as a candidate gene for both PHS and grain color via GWAS in U.S. winter wheat (Lin et al., 2016). Similarly, a study of European winter wheat has recently identified genetic loci for PHS close to both *TaMYB10‐A1* and *TaMYB‐B1* (Scott et al., 2021). While the correlation between grain color and PHS has been often reported, how the *R* genes controlling grain color might pleiotropically influence PHS remains unknown (Lin et al., 2016) and could be due to linkage. While two genes in the HFN/PHS/late‐maturity amylase phenotypic trait complex have been map‐based cloned, *Phs1* on chromosome 4A (Torada et al., 2016) and *TaPHS1* on chromosome 3A (Liu et al., 2013), we did not find GWAS hits at these locations indicating that natural genetic variation at these genes does not play a major role in controlling HFN in U.K. wheat.

Protein content QTL have previously been discovered on all wheat chromosomes (reviewed by Kumar et al., 2018), with major QTL encoding for clusters of for high molecular weight glutenin genes located at the *Glu‐A1, ‐B1*, and *–D1* loci on the long arms of the Group 1 chromosomes (e.g., Zhang et al., 2011). Although we identified three genetic loci for protein content were identified on chromosome 1A, none of them are predicted to be located near the *Glu‐A1* locus (based on gene model *TraesCS1A02G317311* at 509 Mbp). The *GRAIN PROTEIN CONTENT‐B1* (*GPC‐B1*) locus on the short arm of chromosome 6B controlling protein content in tetraploid wheat has been shown to be encoded by a NAC transcription factor (*NAM‐B1*; Uauy et al., 2006). While we found a locus on chromosome 6B controlling protein content, its position at 5 Mbp placed it far from *NAM‐B1* (gene model *TraesCS6B02G207500LC* at 134 Mbp), supporting reports that most hexaploid bread wheat lines do not possess the wild‐type functional form of the gene (Mellers et al., 2020; Uauy et al., 2006). Our finding that grain protein content and grain yield are strongly negatively correlated with each other is in agreement with many previous studies (e.g., Groos et al., 2003; Scott et al., 2021). Indeed, of the 10 genetic loci we identified for protein content, three were also found to putatively co‐locate with hits for yield: *PRT_1A*.1/*YLD_1A.1* on chromosome 1A at 4‐30 Mbp, PRT*_2B.1*/*YLD_2B.3* on chromosome 2B at 698 Mbp, and *PRT_6B.1/YLD_6B.1* on chromosome 6B at 1‐4 Mbp. Interestingly, the *PRT_1A*.1/*YLD_1A.1* region has previously been identified as being located within a putative introgression (Sharma et al., 2021). For the PRT*_2B.1*/*YLD_2B.3* QTL pair controlling grain protein content and grain yield, previous studies have identified a multitrait QTL on chromosome 2B just 4 Mbp away at 694 Mbp (*Qmt.tamu.2B.1.1*, for grain yield, spikes per m^2^, 1,000‐grain weight, and test weight. Peak marker: *RFL_Contig3172_1752*; Assanga et al., 2017; Supplemental Table S6). Additionally, two other protein content QTL, both on chromosome 3B, co‐located with GWAS hits for test weight. The first was QTL pair *PRT_3B.2* (at 564 Mbp)/ *TW_3B.2* (at 567–574 Mbp). A relatively large effect QTL for test weight has previously been reported that spans this interval on chromosome 3B (Cabral et al., 2018; Supplemental Table S6). The second QTL pair was *PRT_3B.3* (819–826 Mbp)/*TW_3B.4* (812–816 Mbp), located further towards the distal end of the long arm of chromosome 3B. Interestingly, beneficial alleles at the first of these pairs of loci increased both protein content and test weight. While no grain yield QTL were identified at this location, it is possible that increased test weight comes at a cost of yield, as fewer but larger grain are formed with a higher protein content. However, our analysis of phenotypic correlations found test weight to be weakly positively correlated with protein content, but to have a stronger positive correlation with grain yield. Further studies are needed to determine whether favorable alleles at the *PRT_3B.2/ SW_3B.2* locus can improve both protein and test weight without affecting yield. The remaining five grain protein GWAS hits were located on chromosomes 1A, 2A, 3A, and 3B. Previously identified grain protein content QTL have been identified on all of these chromosomes, with for example a robust QTL identified on the long arm of chromosome 1A in a biparental population of tetraploid wheat (Blanco et al., 2012) estimated to be in a similar location to our GWAS hit *PRT_1A.3* (peak at 598 Mbp) based on anchoring the simple sequence repeat marker *wmc254* to the wheat physical map at 569 Mbp. However, the large genetic and physical intervals of the majority of these hinders further meaningful comparison. As discussed above, two of the five GWAS hits for test weight co‐located with hits for grain protein content. Of the three remaining test weight QTL, *SW_1B.1* does not appear to be present on the wheat/rye 1B/1R translocation, based on the interval determined by Scott et al. (2021). The remaining two GWAS hits, *SW_3B.1* and *SW_3B.3*, are both located on chromosome 3B, with the latter having the largest effect on test weight of all the genetic loci identified in our panel. Notably, we did not identify GWAS hits at the *Glu‐D1* locus encoding high molecular weight glutenin genes. Cross‐referencing the cultivars in our panel with those previously genotyped for *Glu‐D1* (data sourced from CerealsDB, https://www.cerealsdb.uk.net/cerealgenomics/) found that of the 20 cultivars that overlapped, the high ‘5+10′ *Glu‐D1* allele was present in a quarter of these lines (data not shown). The wheat D subgenome has low genetic diversity, and this is reflected in the low D subgenome SNP coverage of the 90k array (Wang et al., 2014; Mellers et al., 2020). Accordingly, as allelic variation at the *Glu‐D1* locus appears likely to be present at sufficient minor allele frequency to be detected via GWAS, we assume that the *Glu‐D1* locus was not identified in our GWAS for protein content due to lack of SNPs in sufficiently close linkage disequilibrium.

Of the four traits investigated here, the largest number of significant genetic loci were identified for grain yield. Many studies have reported QTL have been identified for yield and yield components (e.g., Gegas et al., 2010; Corsi et al., 2021; Liu et al., 2020; Suliman et al., 2021), and to date just two yield component genes have been identified via map‐based approaches: *Grain Number Increase 1* (*GNI‐A1*; Sakuma et al., 2019) and *WHEAT ORTHOLG OF APO1* (*WAPO1*; Kuzay et al., 2019; Muqaddasi et al., 2019). While we did not identify GWAS hits for yield at either of these genes, *YLD_6A.2* (defined within a 3‐cM interval that corresponds to a large physical interval spanning the centromere on chromosome 6A of around 440 Mbp from 101 to 438 Mbp), spans the wheat gene *GRAIN WIDTH 2*, termed *TaGW2‐A*. Located at 238 Mbp, *TaGW2‐A* and has previously been associated with the control of grain size characters via both forward (e.g., Zhai et al., 2018; Corsi et al., 2021) and reverse genetic studies (Simmonds et al., 2016; Wang et al., 2018; Zhang et al., 2018). The most significant yield QTL identified in our panel, *YLD_2B.4* (−log_10_ *P *= 5.56), was located towards the end of the long arm of chromosome 2B at 766 Mbp. To our knowledge, no yield QTL has been reported in this location, and no wheat orthologues of yield or yield component map‐based cloned genes from the related cereal species rice are located nearby. Recently, Fradgley et al. (2019) used gene‐dropping simulations on a panel of genotyped northwestern European wheat cultivars that overlaps with the panel we investigate here, finding evidence of breeder selection at 15 genetic loci across the wheat genome. Interestingly, a third of these loci either co‐locate or are ≤16 Mbp of GWAS hits for yield we identify here, indicating they have been under strong breeder selection: *YLD_1A.2* (GWAS location = chromosome 1A at 544 Mbp, gene‐dropping location = 1A at 557 Mbp based on SNP *BS00032825_51*), *YLD_2A.1* (GWAS = 2A at 12‐24 Mbp, gene‐dropping = 16 Mbp based on SNP *Excalibur_rep_c110303_320*), *YLD_2A.2* (GWAS = 2A at 763‐774 Mbp, gene‐dropping = 751 Mbp based on SNP *BS00023202_5*), *YLD_2B.1* (GWAS = 2B at 16 Mbp, gene‐dropping = 25 Mbp based on SNP *BS00064706_51*), and *YLD_3A.1* (GWAS = 3A at 625‐627 Mbp, gene‐dropping = 643 Mbp based on SNP *BS00038663_51*).Further investigation and independent validation would be required to further study the yield loci identified.

## 5 CONCLUSIONS

Evaluation of seed quality traits from field grown samples is expensive and time consuming. Therefore, the identification of molecular markers linked to these traits in elite wheat cultivars provides valuable information for further research and ultimately for the development of appropriate genetic markers for marker‐assisted breeding. A key limitation of our study is the relatively small size of the association mapping panel (*n *= 150). However, our finding that (a) all four traits had relatively high heritability (*h* ^2^ ≥ .86); (b) power analysis showed we had reasonable power to detect most of the underlying major QTL and at least a proportion of the smaller‐effect QTL; (c) we detected at least one previously described genetic locus per trait; and (d) co‐localizing GWAS hits between related traits agreed with a priori assumptions about their genetic control (e.g., the *PRT_2B.1*/*YLD_2B.3* QTL pair for protein and yield) provides support for the validity of the results and approaches used. Finally, the demonstration of the utility of historic datasets collected during statutory varietal testing paves the way for similar analyses of for wheat and other crops across the many Union for the Protection of plant Varieties signatory countries.

## AUTHOR CONTRIBUTIONS

Jon White: Data curation; Formal analysis; Investigation; Methodology; Writing – original draft. Rajiv Sharma: Formal analysis; Investigation; Methodology; Writing – review & editing. David Balding: Conceptualization; Funding acquisition; Supervision; Writing – review & editing. James Cockram: Formal analysis; Resources; Writing – original draft; Writing – review & editing. Ian J. Mackay: Conceptualization, Funding acquisition, Project administration, Supervision, Writing‐review & editing.

## CONFLICT OF INTEREST

The authors report no conflicts of interest.

## Supporting information


**Supplemental Table S1**. The 150 United Kingdom (UK) winter wheat cultivars for which 3 yr worth of historical trial data was available, listing best linear unbiased estimates (BLUEs) for the four phenotypic traits investigated. AFP, Application for Protection (a unique identifier used within the varietal registration system); HFN, Hagberg falling number; NA, missing data. ^†^Recommended List. ^‡^Classified as hard (H) or soft (S) grain
**Supplemental Table S2**. Models used to estimate best linear unbiased estimates (BLUEs). Year, year of harvest; TW, test weight; HFN, Hagberg falling number. Management is a cofactor indicating whether fungicide had been applied to the trial or not. TrialID identifies the trial site within the year. ^*^Variety is a fixed term.
**Supplemental Table S3**. Phenotypic correlations (below the diagonal) and their corresponding significance values (above the diagonal, in italic). HFN, Hagberg falling number; TW, test weight; Protein, grain protein content.
**Supplemental Table S4**. Power analysis for the association mapping panel used in this study (*n* lines = 150, *n* mapped markers = 16,801). Analyses were based on quantitative trait loci (QTL) with heritability (*h* ^2^) ranging from .01 to .4. Linkage disequilibrium (LD) between markers and QTL range from perfect linkage disequilibrium (*R* ^2 ^= 1) down to *R* ^2 ^= .01.
**Supplemental Table S5**. Results of genome‐wide association studies (GWAS) using 149 wheat lines for the traits Hagberg falling number (HFN), grain protein content (PRT), test weight (TW), and grain yield (YLD). SNP, single nucleotide polymorphism. Genetic markers that are unmapped are listed as belonging to chromosome '8K', in an arbitrary order.
**Supplemental Table S6**. Summary of significant genome‐wide association studies (GWAS) hits (−log10*P* ≥3, highlighted in pink) when correcting for population substructure using a kinship matrix (KIN), KIN+principal component analysis (KIN+PCA), and KIN+ 1B/1R translocation phenotype used as a co‐factor (KIN+1B1R). HFN, Hagberg falling number; PRT, grain protein content; TW, test weight; YLD, grain yield; NA, not applicable.
**Supplemental Table S7**. Details of the previously published quantitative trait loci (QTL) listed in the Discussion as putatively overlapping with our genome‐wide association studies (GWAS) hits. Physical map positions (Mbp) are based on the reference wheat genome assembly Chinese Spring, RefSeq v1.0 (IWGSC, 2018). NA, not available.
**Supplemental Figure S1**. Quantile‐Quantile (QQ) and histogram plots of the genome‐wide association analysis for Hagberg falling number (HFN; a, e), protein content (b, f), and test weight (c, g). Data for yield using the same collection of cultivars previously published by Sharma et al. (2021) are shown here for comparison (d, h).
