# Supplementary material for: Genome‐wide association mapping of Hagberg falling number, protein content, test weight, and grain yield in U.K. wheat
Source: Crop Sci. 2022 Mar 4;62(3):965–81. doi: 10.1002/csc2.20692 (PMC9314726; doi:10.1002/csc2.20692)
Supplement: Supplementary file 1 — Supplemental Table S1. The 150 United Kingdom (UK) winter wheat cultivars for which 3 yr worth of historical trial data was available, listing best linear unbiased estimates (BLUEs) for the four phenotypic traits investigated. AFP, Application for Protection (a unique identifier used within the varietal registration system); HFN, Hagberg falling number; NA, missing data. †Recommended List. ‡Classified as hard (H) or soft (S) grain Supplemental Table S2. Models used to estimate best linear unbiased estimates (BLUEs). Year, year of harvest; TW, test weight; HFN, Hagberg falling number. Management is a cofactor indicating whether fungicide had been applied to the trial or not. TrialID identifies the trial site within the year. *Variety is a fixed term. Supplemental Table S3. Phenotypic correlations (below the diagonal) and their corresponding significance values (above the diagonal, in italic). HFN, Hagberg falling number; TW, test weight; Protein, grain protein content. Supplemental Table S4. Power analysis for the association mapping panel used in this study (n lines = 150, n mapped markers = 16,801). Analyses were based on quantitative trait loci (QTL) with heritability (h 2) ranging from .01 to .4. Linkage disequilibrium (LD) between markers and QTL range from perfect linkage disequilibrium (R 2 = 1) down to R 2 = .01. Supplemental Table S5. Results of genome‐wide association studies (GWAS) using 149 wheat lines for the traits Hagberg falling number (HFN), grain protein content (PRT), test weight (TW), and grain yield (YLD). SNP, single nucleotide polymorphism. Genetic markers that are unmapped are listed as belonging to chromosome '8K', in an arbitrary order. Supplemental Table S6. Summary of significant genome‐wide association studies (GWAS) hits (−log10P ≥3, highlighted in pink) when correcting for population substructure using a kinship matrix (KIN), KIN+principal component analysis (KIN+PCA), and KIN+ 1B/1R translocation phenotype used as a co‐factor (KIN+1B1R [file CSC2-62-965-s002.docx]

**Supplemental Text 1.** Custom scripts used for power calculations.

R-functions for Power calculations based on the Wang and Xu (2019) Heredity <https://doi.org/10.1038/s41437-019-0205-3>

These are the set of functions for Power calculations as given in the above mentioned publication:

n0<-function(lambda=1,eigvalues=d){

return( (lambda+1)*sum(1/(eigvalues*lambda+1)) )

}

rho<-function(n=500,lambda=1,n0=500){

y<-n0

fn<-function(x,y){

f<-(lambda+1)*( (n-1)/((1-x)*lambda+1) + 1/( (1+n*x-x)*lambda + 1)) - y

return(f)

}

myrho<-uniroot(f=fn,y=y,lower=0,upper=1)

return(myrho$root)

}

power<-function(n=500,h2=0.05,lambda=1,rho=0.5,m=1e5,alpha=0.05){

alpha<-alpha/m

x<-qchisq(1-alpha,1)

n0<-(n-1)/((1-rho)*lambda+1) + 1/((1+n*rho-rho)*lambda+1)

delta<-h2/(1-h2)*(lambda+1)*n0

beta<-pchisq(x,1,delta)

power<-1-beta

return(power)

}

heritability1<-function(lambda=1,n0=2000,power=0.85,m=1e5,alpha=0.05){

alpha<-alpha/m

x<-qchisq(1-alpha,1)

delta<-(qnorm(1-alpha/2)+qnorm(power))^2

h2<-delta/(n0+delta)

return(h2)

}

heritability2<-function(n=2000,lambda=1,rho=0.5,power=0.85,m=1e5,alpha=0.05){

alpha<-alpha/m

x<-qchisq(1-alpha,1)

delta<-(qnorm(1-alpha/2)+qnorm(power))^2

n0<-(lambda+1)*((n-1)/((1-rho)*lambda+1) + 1/((1+n*rho-rho)*lambda+1))

h2<-delta/(n0+delta)

return(h2)

}

sample<-function(h2=0.05,lambda=1,rho=0.5,power=0.85,m=1e5,alpha=0.05){

alpha<-alpha/m

y<-(qnorm(1-alpha/2)+qnorm(power))^2

fn<-function(x,y){

n0<-(x-1)/((1-rho)*lambda+1) + 1/((1+x*rho-rho)*lambda+1)

f<-h2*(lambda+1)/(1-h2)*n0-y

return(f)

}

myh2<-uniroot(f=fn,y=y,lower=0,upper=1e8)

return(myh2$root)

}

Here are the set of R-code for Power calculation. Save the above functions as .R and then use following commands to calculate the Power.

source("powerFunction.R")

####Reading the kinship matrix

kinship<-read.csv(file="kinship_Wheat.csv",header=T)

kinship<-as.matrix(kk[,-1])

qq<-eigen(kk,symmetric=T)

d<-qq$values

u<-qq$vectors

write.csv(x=data.frame(d),file="eigenvalues.csv",row.names=F)

eigvalues<-read.csv(file="eigenvalues.csv",header=T)

d<-eigvalues$d

## **n0() function**:

The effective sample size is determined using by the eigenvalues of the kinship matrix with lambda = 1.

n0(lambda=1,eigvalues=d)

## **rho() function**:

It computes the effective correlation coefficient from the sample.

rho(n= actual population size, lambda=1,n0= effective population size)

##**Power() function**:

power(n= actual population size, h2=QTL, lambda=1,rho=rho-value from above, m=number of markers, alpha=value of alpha)

##QTL-heritability using effective sample size

##**heritability()** **function**:

QTL –heritability using effective population size

heritability1(n0= effective population size,m=number of markers,lambda=1, power=power-value for example 0.85,alpha= value of alpha such as 0.001)

## **heritability2() function**:

QTL –heritability using effective correlation

heritability2(n= actual sample size,rho= effective correlation coefficient,m= number of markers,lambda=1, power=power-value for example 0.85, alpha= value of alpha such as 0.05 or 0.001)

##**sample() function**:

Minimum Sample function with desired QTL-heritability

sample(h2=QTL-heritability,rho= effective correlation coefficient,m= number of markers,lambda=1, power=power-value for example 0.85, alpha= value of alpha such as 0.05 or 0.001)
